# A single PCR-sequencing method to establish the frequency of kdr alleles in the stable fly, *Stomoxys calcitrans*: application to seven livestock farms from south of France

**DOI:** 10.1371/journal.pone.0332229

**Published:** 2025-09-16

**Authors:** Mariana Ribeiro Sato, Géraldine Bossard, Guilhem Sempere, Philippe Jacquiet, Christelle Grisez, Geoffrey Gimonneau, Marc Desquesnes

**Affiliations:** 1 State University of Londrina (UEL), Rodovia Celso Garcia Cid, Campus Universitário, Londrina, Paraná, Brazil; 2 Intertryp, IRD, CIRAD, University of Montpellier, Montpellier, France; 3 CIRAD, UMR INTERTRYP, Montpellier, France; 4 National Veterinary School of Toulouse (ENVT), Toulouse, France; 5 IHAP, UMR 1225 INRAE/ENVT, Université de Toulouse, Toulouse, France; 6 Institut Sénégalais de Recherches Agricoles (ISRA), Laboratoire National de l’Elevage et de Recherches Vétérinaires (LNERV), Route du Front de Terre, Dakar, Hann, Sénégal; University of Ibadan Faculty of Science, NIGERIA

## Abstract

The stable fly, *Stomoxys calcitrans,* is a cosmopolite pest causing direct and indirect nuisances on livestock, due to painful bite, harassment, blood despoliation and biological or mechanical transmission of viruses, bacteria and parasites. Its control is mainly based on direct applications of pyrethroid insecticides on livestock, although evidences show limited efficiency due to short term effects and high levels of phenotypic and genotypic resistance. Diagnosis of genetic resistance is currently based on a series of point mutation PCRs showing limitations. Based on fly specimens trapped in southern France, this study aims to establish a new diagnosis method to investigate *kdr* allele frequencies in livestock farms. A total of 144 *S. calcitrans* specimens were collected from seven farms (1 with insecticide usage, and 6 without) and processed individually through a single PCR using newly designed primers amplifying a 340 bp fragment including the mutation site of the voltage-sensitive sodium channel domain II; PCR products were then sequenced. Among the 104 individuals successfully sequenced, the methodology allowed to detect the *wild-type*, *kdr-his* and *kdr* genotypes encoding for leucine, histidine and phenylalanine, respectively. Although the *wild-type* was the most prevalent, resistance alleles were detected in all farms, especially at the veterinary school, where insecticides are commonly used. These results highlight that genotypic insecticide resistance in *S. calcitrans* populations is widespread. This single PCR-sequencing method, simple, cost-effective and reliable, will allow determining prevalence, distribution and resilience of genotypic pyrethroid insecticide resistance, a primary data to support a conversion from chemical vector control toward environmental-friendly strategies.

## Introduction

The stable fly, *Stomoxys calcitrans* (Linnaeus, 1758), is a Dipteran of significant importance to animal and human health [1,2]. Both male and female of *S. calcitrans* exhibit hematophagous behaviour, and may contribute to the spread of a variety of pathogens, including protozoa, such as *Trypanosoma evansi*, *Besnoitia besnoiti*, viruses, like Equine Infectious Anemia Virus, bacteria, like *Bacillus anthracis*, and infectious larvae of nematodes such as *Habronema microstoma* [3,4].

The painful bites caused by the stable fly can cause severe stress in livestock, which results in significant economic losses due to a drop in weight gain and milk production, among other losses. In the United States, studies estimate annual losses ranging from about 840 million to 2.3 billion USD [2]. Meanwhile, in France, annual losses are projected to be 234 million USD for the dairy industry and 145 million USD for the meat industry [5].

Livestock production systems are ideal environments for the viability of the flies’ reproductive cycle [6], as their larvae preferentially develop in decomposing organic matter, such as soiled animal bedding and leftover hay. The wide variety of places where the larvae can develop, along with the flies’ behaviour, briefly visiting the host just to get a blood meal, makes chemical control of these populations quite challenging [7].

Chemical insecticides have been proven ineffective against stable flies on multiple occasions in the past few years [8–11], but their usage is still widespread. Moreover, fly populations have developed genetic resistance as a result of continuous exposure to insecticides [12].

Knockdown resistance (*kdr*) is one of the main genetic mechanisms that reduces the effectiveness of pesticides such as dichlorodiphenyl-trichloroethane (DDT) and pyrethroids in several species of livestock and human pests [13,14]. This resistance arises from mutations in the voltage-sensitive sodium channel (*Vssc*) gene encoding the voltage-gated sodium channel (VGSC) protein, the site of action of these insecticides [15]. Specifically, the substitution of the nucleotide thymine for adenine in codon *kdr* at position 1014 in *S. calcitrans* (exhibiting CAT instead of CTT)*,* causes the amino acid leucine (L) to be exchanged for histidine (H) (L1014H; *kdr-his*) [16]. The alternative mutation, described for the first time by Olafson et al. [17] in *S. calcitrans*, involves the change on this codon, from CTT to TTT, resulting in the exchanged of the amino acid leucine (L) for phenylalanine (F) (L1014F; *kdr*), identified only in samples from the *École Nationale Vétérinaire de Toulouse* (ENVT) southwestern France, and in Thailand. The study concluded that the *kdr* mutation, L1014F, is restricted to Europe and Asia in stable flies, since this allele was not detected in Costa Rica or in ten states of the United States. A recent study, performed in Germany confirmed that the *kdr* allele is present in high frequency (34%) in other places in Europe [18]. They also looked for the *super-kdr* (M918T) polymorphism as described in house flies or horn flies, *Haematobia irritans irritans* L. (Diptera: Muscidae) [19–21] but did not find it in *S. calcitrans*. Therefore, in order to effectively control farm animal pests and implement adapted vector control strategies, it is imperative to identify and comprehend the distribution of such mutations.

In order to investigate *kdr* status of stable flies from some farms in *Occitanie* region, France, in a preliminary study on 42 stable flies from 7 villages (S1 Table), point mutation PCR diagnostics using the protocol and several sets of primers published by Olafson et al. [16,17], and also 3 sets of our own candidate primers designed specifically to amplify separately the 3 *kdr* alleles (coding for Leucine, Histidine and Phenylalanine) were conducted. In both cases, using our primers or the primers previously published, in significant numbers of cases, ranging from 27–71% of the samples, unexpected positive results were obtained with the 3 sets of primers for single fly samples. It was therefore concluded that *kdr* alleles diagnosis based on point mutation PCRs were not reliable, and it was decided to develop a single PCR and direct sequencing approach.

Therefore, this study aims to establish a diagnosis method to investigate the frequency of the various *kdr* alleles in livestock farms, based on fly specimens obtained from seven locations in South of France, through a single PCR and a partial sequencing of the domain II region of the *Vssc* gene.

## Materials and methods

### Collection sites

Flies have been collected from 7 farms located in the villages of Aubiet (Latitude: 43.64, longitude: 0.784), Bellegarde-Marsal (Latitude: 43.907, longitude: 2.274), Castelnau d’Arbieu (Latitude: 43.883, longitude: 0.704), Toulouse (*École Nationale Vétérinaire de Toulouse*/ENVT; latitude: 43.602, longitude: 1.384), Escorneboeuf (Latitude: 43.654, longitude: 0.914), Homps (Latitude: 43.809, longitude 0.85) and Marsan (Latitude: 43.656, longitude: 0.721). Main characteristics of the farms are reported in Table 1. All farms were selected based on the absence of use of insecticide/acaricides for the control of flies or ticks, except the ENVT which was chosen as a positive control, since insecticides are used for years and *S. calcitrans* phenotypic and genotypic resistance were already demonstrated [10,17].

**Table 1:** Main characteristics of the 7 farms involved in the study.

Village names	Aubiet	Belleguarde-Marsal	Castelnau d’Arbieu	Escorneboeuf	Homps	Marsan	Toulouse (ENVT)
Type of production	Meat production (milk suckling)	Meat production (milk suckling)	Milk production	Meat production (milk suckling)	Meat production (milk suckling)	Meat production (milk suckling)	Growing heifers
Cattle breed(s)	*Blonde d’Aquitaine*	*Limousine*	90% *Prim’Holstein* 10% others	*Blonde d’Aquitaine*	*Blonde d’Aquitaine*	*Blonde d’Aquitaine*	*Holstein*
Number of cows	100	85	60	50	50	85	12 heifers + 40 horses
Pasturing	Yes	Yes	Yes	Yes	Yes	Yes	Yes
Useful Agricultural Surface (*SAU*)	120	74	230	93	130	158	ND
Use of insecticides	No	No	No	No	No	No	Yes

### Flies collection

In all farms, a total of 16–30 adults *S. calcitrans* were individually collected using an entomological hand-net, during 3 trapping sessions organised in November 2023, March 2024 and July 2024, at the exception of Bellegarde-Marsal where flies were collected only in July. Collection at three different periods of the years aimed at avoiding any mass-emerging effect (bias) of a specific allele. After collection and species identification, flies were individually transferred to absolute ethanol in Eppendorf tubes and stored at −20°C until further analysis.

### Dissecting and grinding the flies

To avoid any contamination between samples, technicians were wearing gloves; tweezers and scalpel were disinfected between each fly by dipping the dissecting instruments in 2% bleach, followed by immersion in double-distilled water, followed by immersion in 70% alcohol, followed by immersion in double-distilled water and final drying with absorbent paper. Each fly was removed from its container with absolute alcohol and placed on a sheet of absorbent paper using disinfected tweezers; the head was separated from the body using a scalpel, and then individually placed in a 1.5 ml microtube containing two autoclaved stainless-steel spheres (0.7 mm in diameter) and 100 μl of double-distilled water. Microtubes were then processed in the TissueLyser II at 250 rpm for 30 seconds. After grinding, 90µl of the supernatant were transferred to a new 1.5 ml microtube, carefully avoiding to suck the beads.

### Resin preparation

Genomic DNAs were prepared using the Chelex 100 method (Chelating Ion Exchange Resin, Biorad ref 143–2832, 100g), as described by Walsh et al. [22]. Initially, two water baths were preheated to 56°C and 95°C. Then, 100 μl of 5% Chelex 100 was transferred to the microtube containing the 90 μl crushed fly head supernatant. After vortexing, the tube was incubated for 1 hour at 56°C and then for 30 minutes at 95°C. The supernatant of this preparation was further used for PCR.

### Primer design

Two primers were designed using the software “Primer 3 web” (Version 4.1.0) based on the sequence of *S. calcitrans Vssc* gene obtained from GenBank (accession number HQ010283.1), requesting a PCR product >300 bp and including the mutation site of the *Vssc* domain II (Amino acid 1014).

### Sodium channel domain II gene amplification

A part of the *Vssc* domain II gene was amplified using a primer pair designed as indicated above, and PCR conditions described in Table 2. A premix was prepared containing 17.4 µL of H₂O, 2.5 µL of 10X PCR buffer, 0.5 µL of 10 mM dNTPs, 1 µL of each primer (10 µM), and 0.1 µL of Taq DNA polymerase (5 U/µL), totalling 22.5 µL per tube. Next, 2.5 µL of the supernatant from each Chelex-prepared sample was added to the premix in its respective well in the PCR plate, leading to a final volume of 25 µL per reaction. After PCR thermocycling (see Table 2 for cycling conditions), PCR products were loaded under 10 µL with 5 µL blue load (50 ml Glycerol; 1 ml EDTA 100mM; 0.4% (w/v) bromophenol blue; 0.4% (w/v) xylen cyanol; qsp 100 ml bi-distilled water) in a well of 2% agarose gel. The DNA migration was done under 110 volts for 2 hours.

**Table 2:** Oligonucleotide primer sequences used to amplify a part of the Vssc domain II of *S. calcitrans.*

Primer names	Primer sequences (5’-3’)	Target	Cycling conditions
F0994	5’ AGAGTCCATGTGGGACTGTATG 3’	Vssc Domain II, Expected size 340	95ºC, 5 min; 30 cycles (95ºC, 30s; and 60ºC, 30s; and 72ºC, 1 min); 72ºC, 5 min
R1053	5’ TCACCCAGTTCTTAAAACGAGA 3’		

### DNA sequencing

After amplifying part of the *Vssc* domain II, the PCR products and the primer sequences were sent for double sense sequencing (Sanger sequencing) at the pharmaceutical company Eurofins CDMO (Contract Development & Manufacturing Organization). The data obtained from the sequencing were visualized using AliView software [23]. The chromatograms were used to carefully check the sequences and to detect mutations in heterozygote flies, which allowed to identify the nucleotide substitution(s) responsible for resistance in flies. Final sequences were reconstituted indicating homozygotes or heterozygotes status of the flies DNA analysed. Frequency of resistant alleles and genotypes were established.

### Statistical analyses

Hardy–Weinberg equilibrium (HWE) was tested on the observed population genotypes using the Tri-allelic Exact test for HWE (HWTriExact) [24]. The HWE was calculated based on the assumption that the *kdr* mutations are located on an autosomal gene. In association, we performed pairwise comparisons tests for proportions to compare allelic frequencies between farms. In this test, wild allele (L) frequency was compared to resistant ones (H + F). R software (version 4.4.1) was used for all statistical analyses [25].

## Results

Sequence of the primers designed using Primer3, F0994 and R1053, are presented in Table 2. The size of the PCR product expected is 340 bp. Hybridization sites of the primers are located in highly conserved regions of the gene [26].

**Fig 1:**
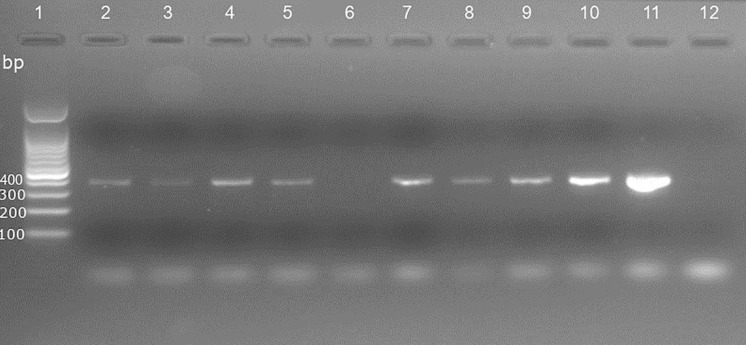
Visualization of PCR products obtained using primer F0994/R1053 amplifying a part of the *Vssc* domain II of *S. calcitrans.* Legend: lane 1: Molecular marker (Bench top ladder 100pb, Promega, Madison, United state), lanes 2-4: Marsan samples n° 8, 9 & 10; lanes 5-10: Toulouse samples n° 3, 4, 5, 6, 7 & 9; lane 11: positive control; lane 12: negative control.

**Fig 2:**
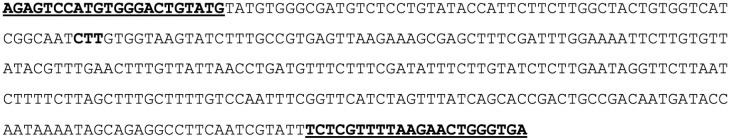
Predicted 340 bp sequence using primers F0994 and R1053 for amplification of a part of the Vssc domain II gene of a wild fly DNA. Hybridization sites of the primers F0994 and R1053 are underlined in bold; nucleotides of the codon 1014 are in bold with * (the sequence CTT of the wild fly, is coding for Leucine); in italics are the nucleic acid of the intron (other nucleic acids, in blocs of 3 letters, are codons of the sodium channel protein).

A total of 144 *S. calcitrans* was collected in the seven farms. After DNA extraction, 118 (82%) were successfully amplified (see Fig 1 for visualization of PCR product migration, S1 Raw Images for all PCR product migration and S2 Table the related sample database), and 104 (88%) underwent successful sequencing; in all, around 72% of the fly specimens was successfully sequenced. A representative set of the sequences obtained was submitted to GenBank and available in supplementary information (S3 Table). An example of the predicted product (showing the wild-type allele: CTT) is given on Fig 2. Overall, a minimum of 13 sequences were obtained from each of the 7 farms sampled (S4 Table).

**Fig 3:**
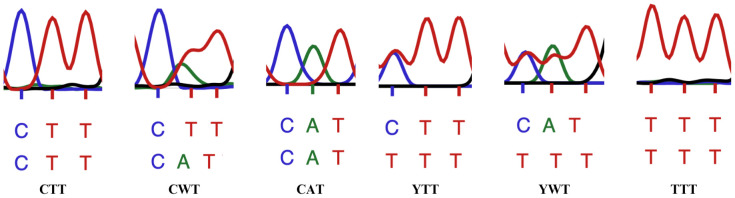
Extracts of the 6 types of chromatograms obtained for codon 1014 from stable fly, *S. calcitrans* by sequencing. Each nucleotide is represented by a colour: Cytosine/blue, Thymine/red and Adenine/green; peaks of the chromatogram curves indicate the type of nucleotide present at this location. The 3 lines below the chromatograms are from the top to the bottom: First line (coloured block letters): sequencing results indicated by the sequencing machine, based on the highest peaks. Second line (coloured block letters): sequencing results indicated by the secondary peak (heterozygoty) when present. Third line (black block-letters): sequence (homozygoty) or coded sequences (heterozygoty) obtained. For example, in the second codon from left: the first line indicates dominant nucleotides CTT (the red line, for Thymidine, is the highest in the 2 last locations), the second line indicates CAT (the green line is almost equally high in the second location); therefore, this codon reveals heterozygoty and the second nucleotide, T or A, is coded as “W”, in black colour, on the third line, showing “CWT”. W means A or T; Y means C or T.

Amongst 104 sequences obtained it was possible to detect the 6 following chromatograms:

(1) CTT (wild-type), (2) C(A/T)T (noted CWT), (3) CAT, (4) (C/T)TT (noted YTT), (5) (C/T)(A/T)T (noted YWT) (in the present case, only 2 combinations of YWT are possible: CAT and TTT), and (6) TTT. Representation and interpretation of these chromatograms are shown in Fig 3.

From sequencing results, the *kdr* codons 1014 of PCR products obtained using primers F0994 and R1053 are presented in supplementary material (S3 Table). The frequency of *kdr* alleles and genotypes found in 104 flies successfully sequenced, from the 7 farms investigated are presented in Table 3. Results revealed that the 3 known allelic sequences of the targeted triplet coding for Leucine (CTT, *wild type*), histidine (CAT, *kdr-his*), and phenylalanine (TTT, *kdr*) are all present in *S. calcitrans* populations trapped in seven locations of South of France. The *wild-type* allele (Leucine) was detected at the highest frequency (from 0.62 in Homps to 0.93 in Castelnau d’Arbieu and Marsan) in all farms, except at the national veterinary school of Toulouse (*ENVT*, 0.25). The *kdr-his* allele was the second most detected, also present in all farms, ranging from 0.03 in Castelnau d’Arbieu to 0.38 at the *ENVT*. The *kdr* allele was detected in only 4 farms, at a very low frequency (0.03 to 0.08), except at the *ENVT* where it was detected at 0.38. The genotype frequencies of all farms were not significantly different from the expectations of the Hardy–Weinberg equilibrium (p > 0.05, Table 3). Pairwise comparison of wild and resistant alleles between farms highlight that resistant alleles were significantly more frequent a ENVT (p < 0.05) than all farms except for Homps (p = 0.321, S4 Table).

**Table 3:** *Kdr* allele and genotype frequencies in 104 stable flies collected from 7 locations in Occitanie, south of France.

Location	Allele frequency				Genotype frequency						Exact test HWE
	N	L	H	F	LL	LH	HH	LF	FH	FF	p-value
Aubiet	18	0.81	0.19	0	0.67	0.28	0.06	0	0	0	0.512
Bellegarde-Marsal	14	0.75	0.21	0.04	0.57	0.29	0.07	0.07	0	0	0.347
Castelnau d’Arbieu	15	0.93	0.03	0.03	0.87	0.07	0	0.07	0	0	0.965
Toulouse (ENVT)	16	0.25	0.38	0.38	0	0.38	0.13	0.13	0.13	0.25	0.099
Escorneboeuf	13	0.88	0.12	0	0.85	0.08	0.08	0	0	0	0.120
Homps	13	0.62	0.31	0.08	0.38	0.46	0.08	0	0	0.08	0.057
Marsan	15	0.93	0.07	0	0.87	0.13	0	0	0	0	0.965
All farms	104	0.74	0.19	0.07	0.60	0.24	0.06	0.04	0.02	0.05	na

N, number of samples sequenced; L, leucine; H, histidine; F, phenylalanine.

## Discussion

Due to unreliable point mutation PCR results obtained in preliminary works using the 3 sets of primers described by Olafson et al. [16,17] or derived ones (S1 Table), the objective of the present study was to establish a simple diagnosis method based on amplification through a single PCR, and sequencing of a part of the domain II region of the *Vssc* gene, to investigate the frequency of *kdr* alleles in *S. calcitrans* and to apply this method to fly specimens obtained in seven locations in *Occitanie*, South of France. Results indicate that this method is efficient and highlight the presence of *kdr* and *kdr-his* alleles in stable fly populations in 6 locations of the South of France, on top of the ENVT (Toulouse) which had already been described [17].

Using a primer set developed in this study (F0994, R1053; Table 2), a 340 bp sequence part of the *Vssc* gene encoding the voltage-gated sodium channel protein has been successfully amplified and sequenced in 104 flies out of 144 (72%); various reasons may explain failures. Wild fly specimens caught may be carrying various types of PCR inhibitors that the Chelex procedure may not neutralize; this may lead to poor or nil PCR products; in the present case, only 18% of the samples provided nil or low PCR products and were therefore not sent for sequencing; whether these 26 samples would have provided readable sequences is not known, but, they would most probably not be suitable for sequencing. When considering the sequencing step only, 12% of the 118 samples submitted for sequencing failed to provide complete readable and publishable sequences; reasons for failure are classically linked with the sequencing conditions; some rare products were totally missing (sequencing inhibitors, contamination, DNA destruction…), while most of them provided truncated sequences, and were therefore not included in the acceptable results. Overall, 88% of sequencing were successful, which sounds acceptable. Selection of strong PCR products for sequencing may improve this rate but could also introduce a bias that we preferably avoided. A polymorphism in the amplified portion of the *Vssc* gene could be at the origin of negative PCR results, however, within a highly conserved gene [26], this is of low probability; indeed, as obtained from Genbank, F0994 and R1053 exhibit 100% identity with *Stomoxys*, *Haematobia* and *Musca* (consultation Skyblast 11 April 2025: https://sky-245blast.com/blast/n/cab4380e09e4 & https://sky-blast.com/blast/n/1ad58a4c2095). It is therefore advisable to oversize the target sample size of at least 25% for further epidemiological studies. Chromatograms of the 104 sequences obtained unambiguously provided classification of the fly genotypes. There was no doubtful interpretation, as was observed in the preliminary studies using point mutation PCRs (sometime indicating 3 different alleles for one fly). In a somehow similar approach, other authors recently proposed an RT-PCR method allowing to distinguish homozygoty and heterozygoty for *kdr* alleles; in their model, the relative value of the Cq obtained with 3 specific primer-sets was interpreted to conclude on the genotype; although it was most of the time possible, some samples remained uninterpretable [18].

Results of our study showed the three types of polymorphisms in the *kdr* codon, i.e., the wild-type CTT coding for leucine, the *kdr-his* genotype CAT coding for histidine and the *kdr* genotype TTT coding for phenylalanine were found in *S. calcitrans* trapped in seven locations of South of France, highlighting that insecticide resistance seems widespread. In all the farms sampled and especially the 6 where insecticides are not used, the *kdr-his* mutation was found in each of them, and the *kdr* mutation was found in three of them, indicating possible spreading of resistant flies from neighbouring farms, or remanence of resistant alleles in the fly population; hypotheses that would need further and larger sample size investigations. Nevertheless, except for ENVT, the wild type remains the most prevalent allele (62% to 93%) and homozygote wild-type flies were abundant (38% to 87%) in farms where insecticides are not used. On the opposite, ENVT is the only location where the wild allele is minor, with an allelic frequency of 16% and a total absence of homozygote wild-type flies. Indeed, it is known that the frequent use of an insecticide (pyrethroids in the present case) exerts a strong selective pressure, stimulating the spread of resistance alleles in flies such as in *S. calcitrans* populations, but also a wide range of insect species [27,28]. In comparison to the other 6 locations where insecticides are not used on cattle, the higher frequency of mutated alleles observed at ENVT may be linked with a high pressure of insecticide-use requested at veterinary school for various reasons, including (i) the control of the high density of *S. calcitrans* as a consequence of a large suitable larval habitat, (ii) regular and frequent entrances of sick animals for clinical care which, as a consequence, generates the need to prevent pathogen spreading by mechanical vectors, and the (iii) presence of animals that need to be kept strictly free of pathogens for several reasons in links with veterinary teaching and research.

This regular exposition to insecticides of the *Stomoxys* population at ENVT led to the highest frequency of the *kdr-his* and *kdr* mutation, both present at equal frequencies (0.38), and with the highest frequency of homozygotes *kdr*-type flies (0.25). This observation of equal level *kdr-his* and *kdr* mutation in a population was also made in Germany [18]. Results obtained in this study are substantially different from those reported by Olafson et al. [17] in terms of allele and genotype frequency at ENVT; in their study on 30 samples, the *kdr* allele was predominant with 85% (38% in our study) and the homozygote genotype FF reached a frequency of 80% (with a limited heterozygoty: 0 LF and 10% FH), while we observed respectively 25%, 13% and 13%. Conversely, for *kdr-His*, while they observed only 7% homozygoty and no heterozygoty, we observed respectively 13% and 38%. These important variations in genotype frequencies between these two studies could be due to seasonal effects and insecticides usage at a specific period of the year. Our study may also better reflect the whole population genotypes since flies were trapped in 3 different occasions along 9 months (November 2023, Mars and July 2024). The genotype frequencies of all stable fly populations examined were not different from the expectations of the Hardy–Weinberg equilibrium, which suggests that no selection process regarding insecticide resistance is ongoing in these populations. Even if a larger sampling size would better support our conclusions, these small size survey results are in agreement with the farming systems. Indeed, in the 6 farms without insecticides, no ongoing selection is expected, and at ENVT, on the opposite, insecticides are regularly used for decades, selection has probably been done for long.

Overall, the results of the genetic characterisation of resistance is congruent with previous phenotypic evaluations of resistance performed on *S. calcitrans* populations in the southwest of France, a geographically similar region to the current study [10,11]. Authors exposed two laboratory strains, a sensitive strain (no more available at the present time) and a resistant strain from ENVT, and wild flies from several farms to a range of insecticide products, at the recommended doses proposed by the manufacturers, and showed that none of the populations (except the sensitive one) was fully susceptible, suggesting the presence of insecticide resistance mechanisms. This hypothesis was further on validated by Olafson et al.‘s (2019) who detected the presence of the L1014F mutation in *S. calcitrans* from ENVT. Our results presented here demonstrate that *kdr* mutations are not confined to the *Stomoxys* population of the ENVT but widely distributed in the region and probably in the whole country; this situation needs to be explored. In Germany, in an area neighbouring France, a recent study performed on *Stomoxys* from dairy farms highlighted the high frequency and distribution of *kdr* mutations (34%) [18].

Presence, distribution and frequency of these resistant alleles, as well as other potential mechanisms of resistance to pyrethroids, should be further explored in livestock breeding areas to better evaluate the potential of chemoresistance in reducing the interest and cost effectiveness of such insecticide treatments. Even more, once chemoresistance is established, not only the use of insecticides/acaricides is a loss of money, but it is also a way to contaminate the environment, affect biodiversity, introduce residues in animal and human food, and, possibly kill useful actors and bioregulators such as dung beetles (which provide many ecosystemic services) [29,30] and some of their phoretic mites such as *Macrocheles* spp. (which may reduce *Haemonchus contortus* pressure while feeding on their larvae) [31].

The single PCR and sequencing method presented here is quick and cost-effective; the cost is estimated around 5€ per fly, therefore 50–75€ per farm (fly trapping not included). Besides, using a biological diagnosis method such as the “flybox” [9] would be a complementary method to this pure laboratory technique, and would bring phenotypic information to complete genotyping. Overall, due to the spreading of *kdr* alleles, and more generally insecticide resistance, alternative control methods to insecticides, such as vegetable waste management, insect trapping and the use of micro-predators of larval stages should be explored and favoured.

In conclusion, this individual fly single PCR-sequencing method proved efficient to investigate the frequency of *kdr* alleles in *Stomoxys*, and could possibly be extended to other flies such as *Haematobia* and *Musca*. *Kdr* alleles were found in all farms sampled, even in the absence of insecticides usages, suggesting that chemical resistance in *Stomoxys* is widespread and questioning about direct effect and side-effect of the use of insecticide on farms in France, as well as in the resilience of these mutations in fly populations. Future studies should extend the scope of the study to the whole of France, screening stable fly populations for the frequency of resistance alleles as a predictor of phenotypic resistance. This data will be of significant value to promote alternative vector control methods, respective of the environment and global health.

## Supporting information

S1 Table Point mutation PCR results“ using Olafson et al 2019 and our candidate primers for detection of three different kdr alleles (Leucine, Histidine and Phenyl-alanine) and percentage of “triple positive” results obtained in 42 individual stable fly samples. Letters in the fly reference are indicating the geographical locations; A: Aubiet, C: Castelnau d’Arbieu, E: Escorneboeuf, H: Homps, L: Lamayoux, M: Marsan, T: Toulouse; numbers are serial numbers of the fly specimens; for PCR results: 1 = positive, 0 = negative; for Genotypes: L = Leucine, H = Histidine & F = Phenylalanine.
(XLSX)

S2 Table PCR results of 144 stable flies from 7 locations of Occitanie region, South of France. (XLSX)

S3 Table Single fly *kdr* genotypes of stable flies obtained from partial sequencing of *Vssc* Domain II, using F0994 and R1053 primers (Expected size 340 bp) and their and DNA sequences (to be published in GenBank). (XLSX)

S4 Table Fly references and their codon 1014 nucleotide sequences (coding for amino acid 1014) at the kdr mutation site, of 104 stable flies from 7 locations of Occitanie region, South of France. (XLSX)

S5 Table Matrix of pairwise comparisons between wild allele (L) and resistant ones (H + F) frequencies between localities with correction (bonferroni) for multiple testing. (XLSX)

S1 Raw Images Original gel images of PCR product migration of 144 *Stomoxys* samples. Pictures were taken with Gel Imager bio, Vilber Lourmat. Samples were loaded from the left side to the right side. Molecular weight marker was denoted “MM”, positive controls “C+”, negative controls “C-”, and all samples were annotated from 1 to 144 according to their numbering in the database S3 Table.
(PDF)
