# Supplementary figures and images for: A single PCR-sequencing method to establish the frequency of kdr alleles in the stable fly, Stomoxys calcitrans: application to seven livestock farms from south of France
Source: PLoS One. 2025 Sep 16;20(9):e0332229. doi: 10.1371/journal.pone.0332229 (PMC12440155; doi:10.1371/journal.pone.0332229)

MM 1 2 3 4 5 6 7 8 9 10 11 12 13 14 15 16 C- C+

bp

400  
300  
200  
100

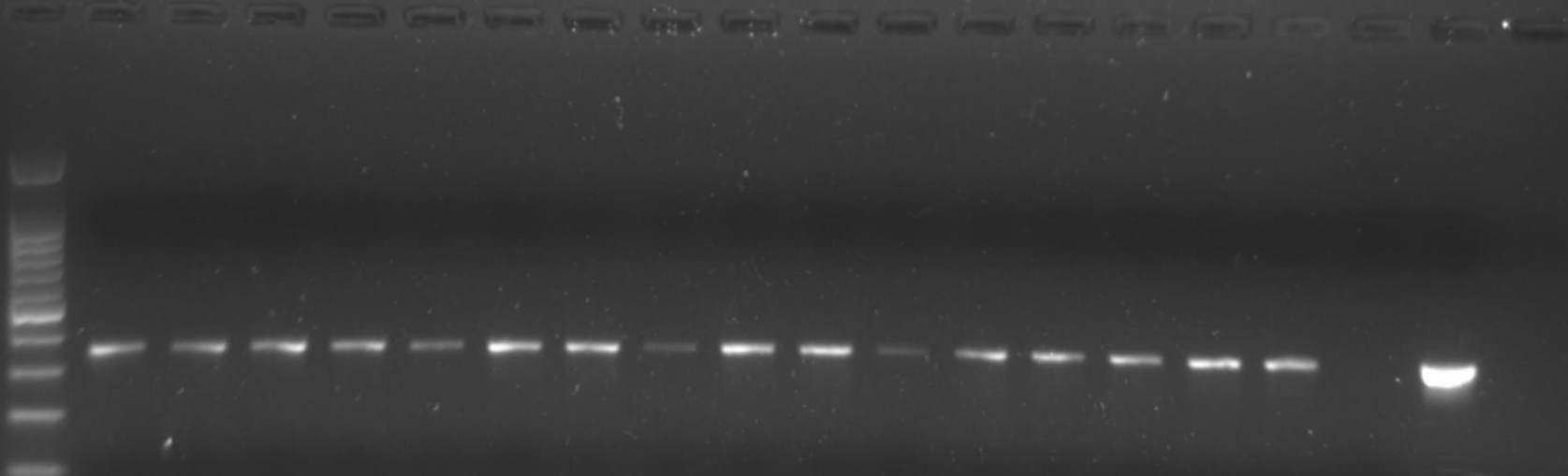

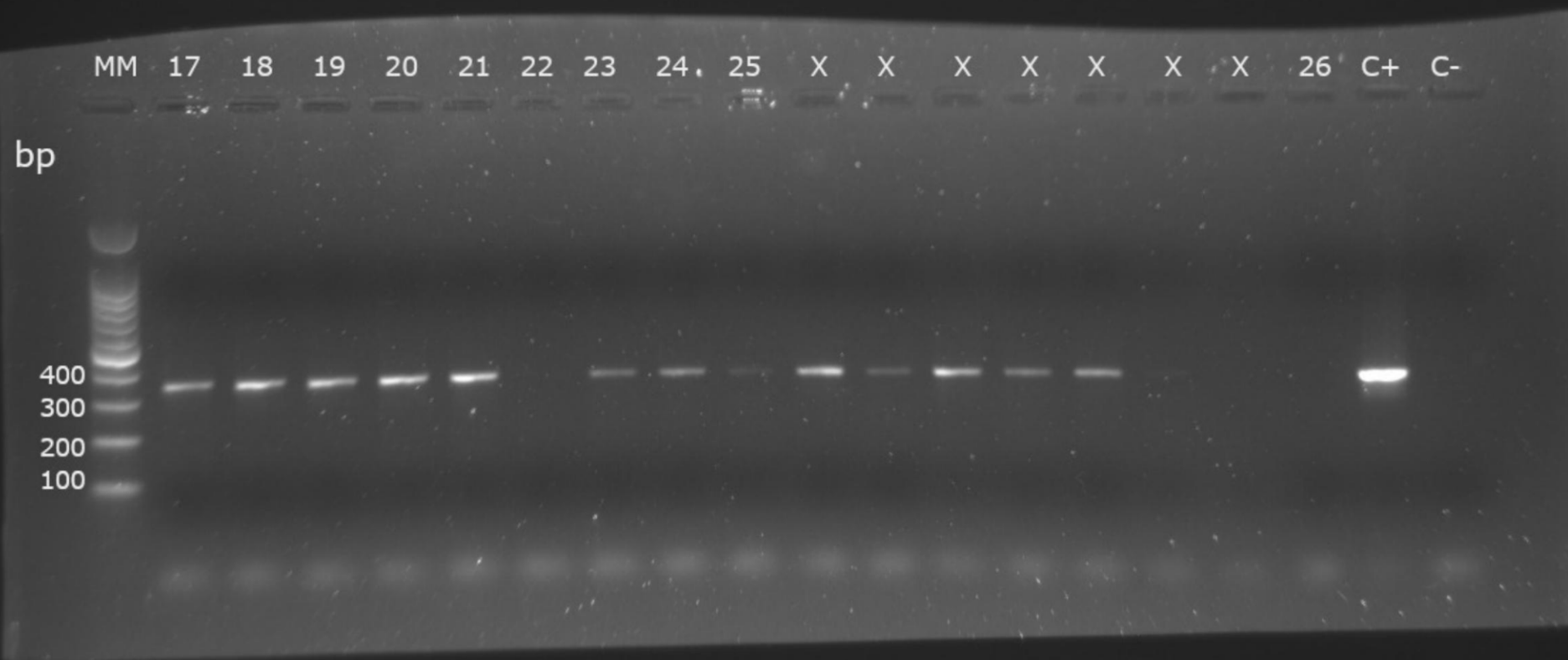

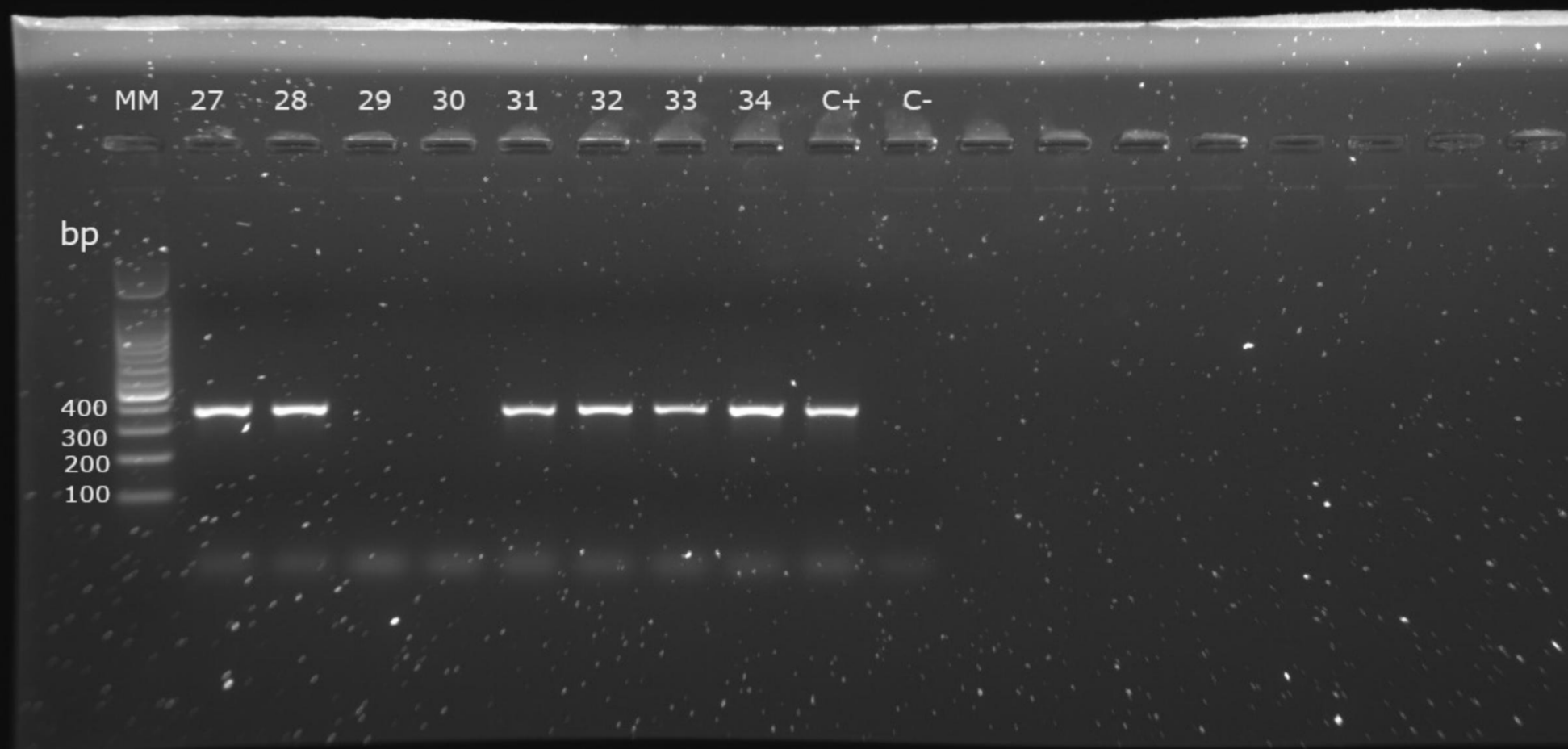

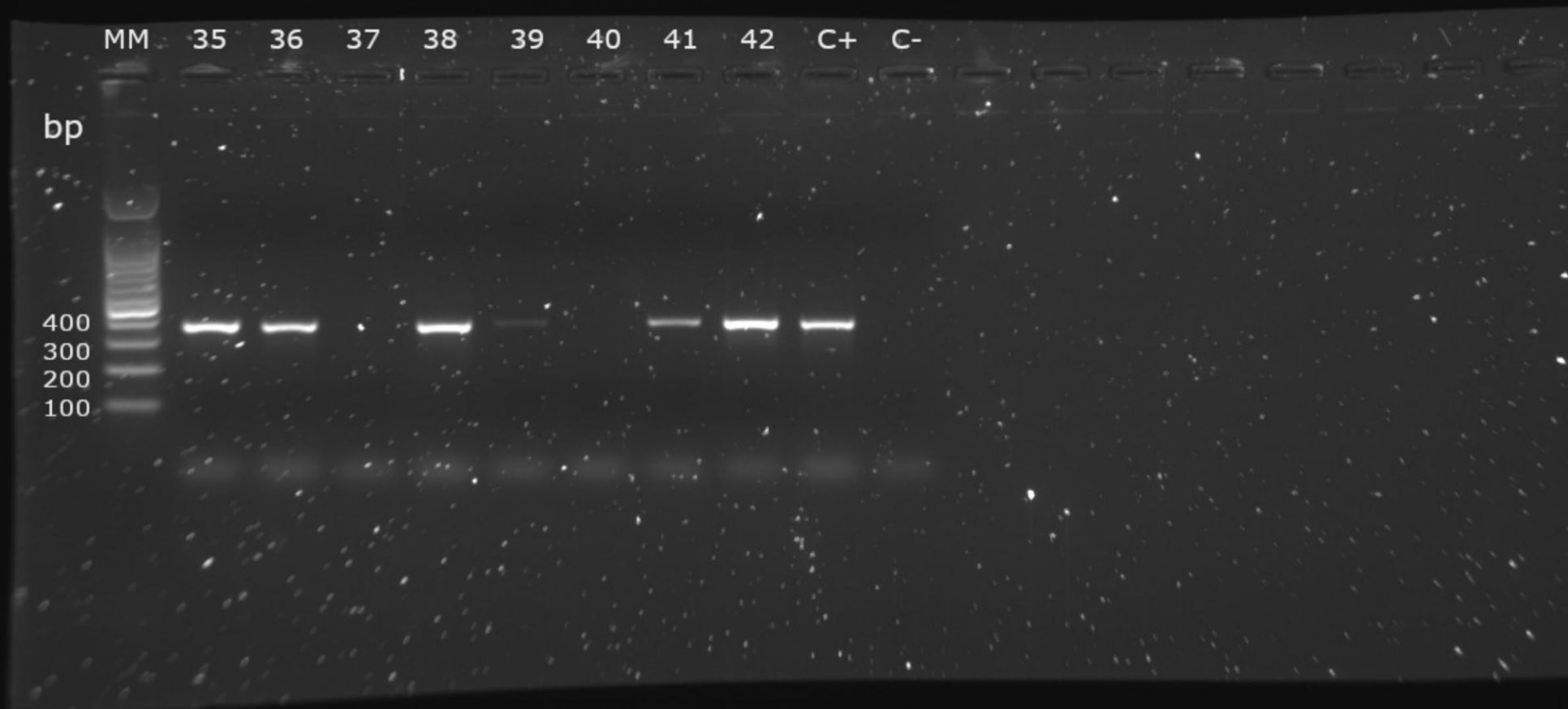

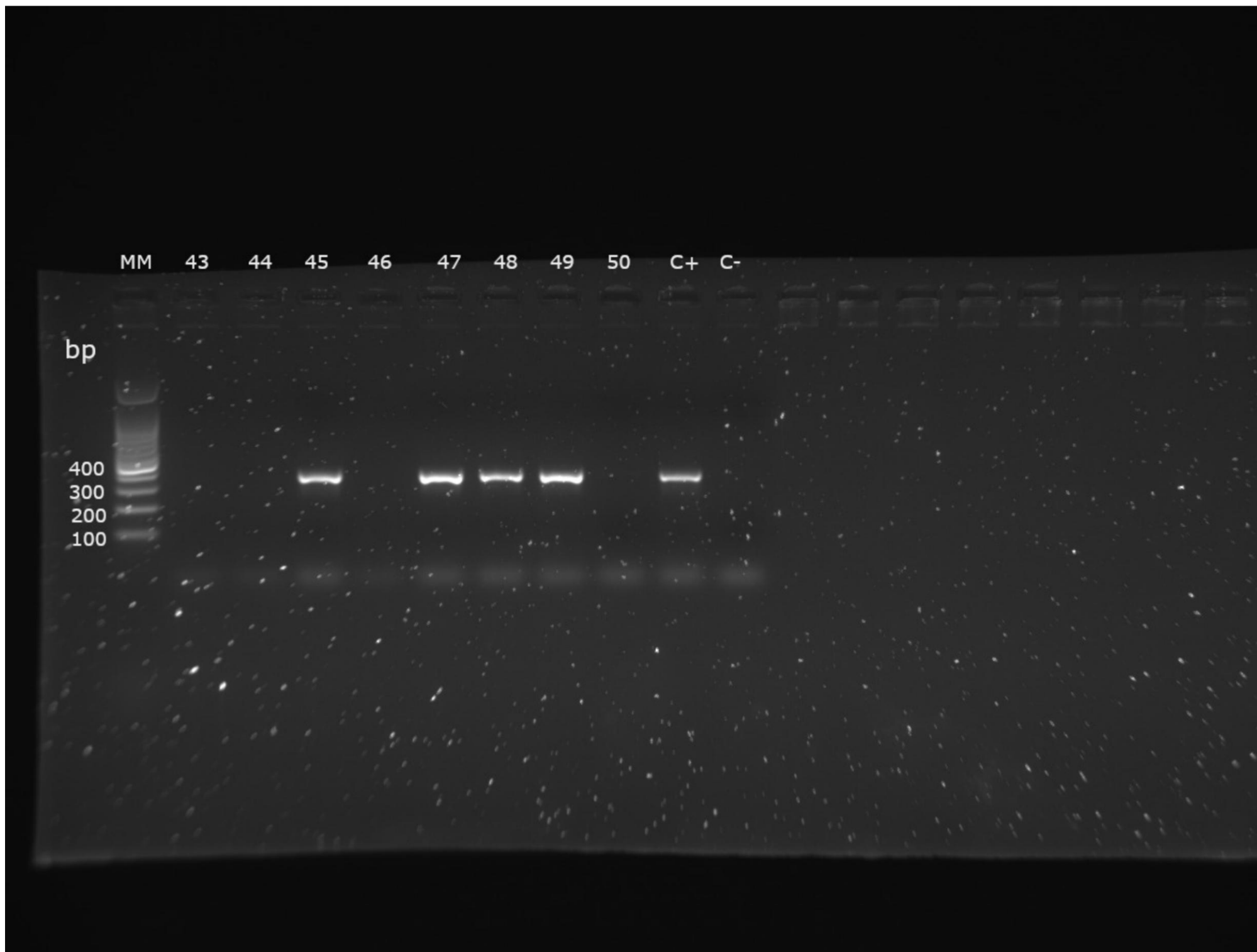

MM 51 52 53 54 55 56 57 58 59 60 61 62 63 64 65 66 C+ C-

bp

400  
300  
200  
100

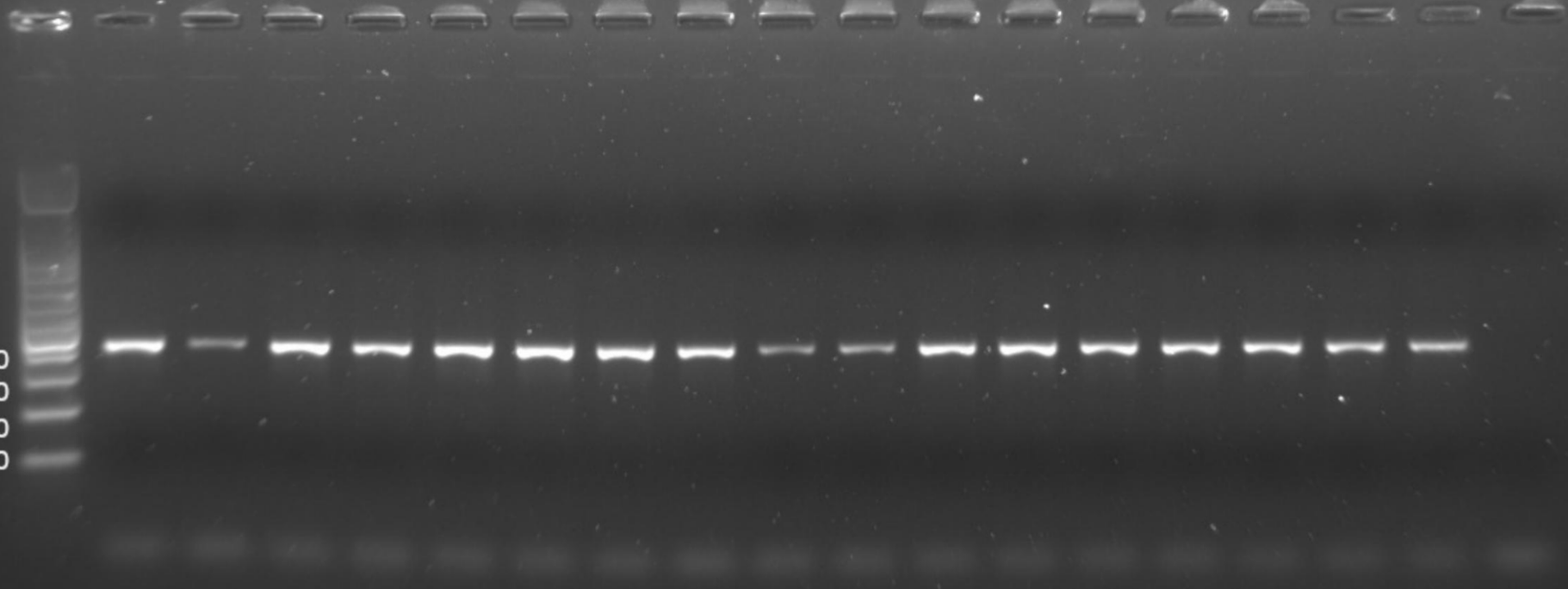

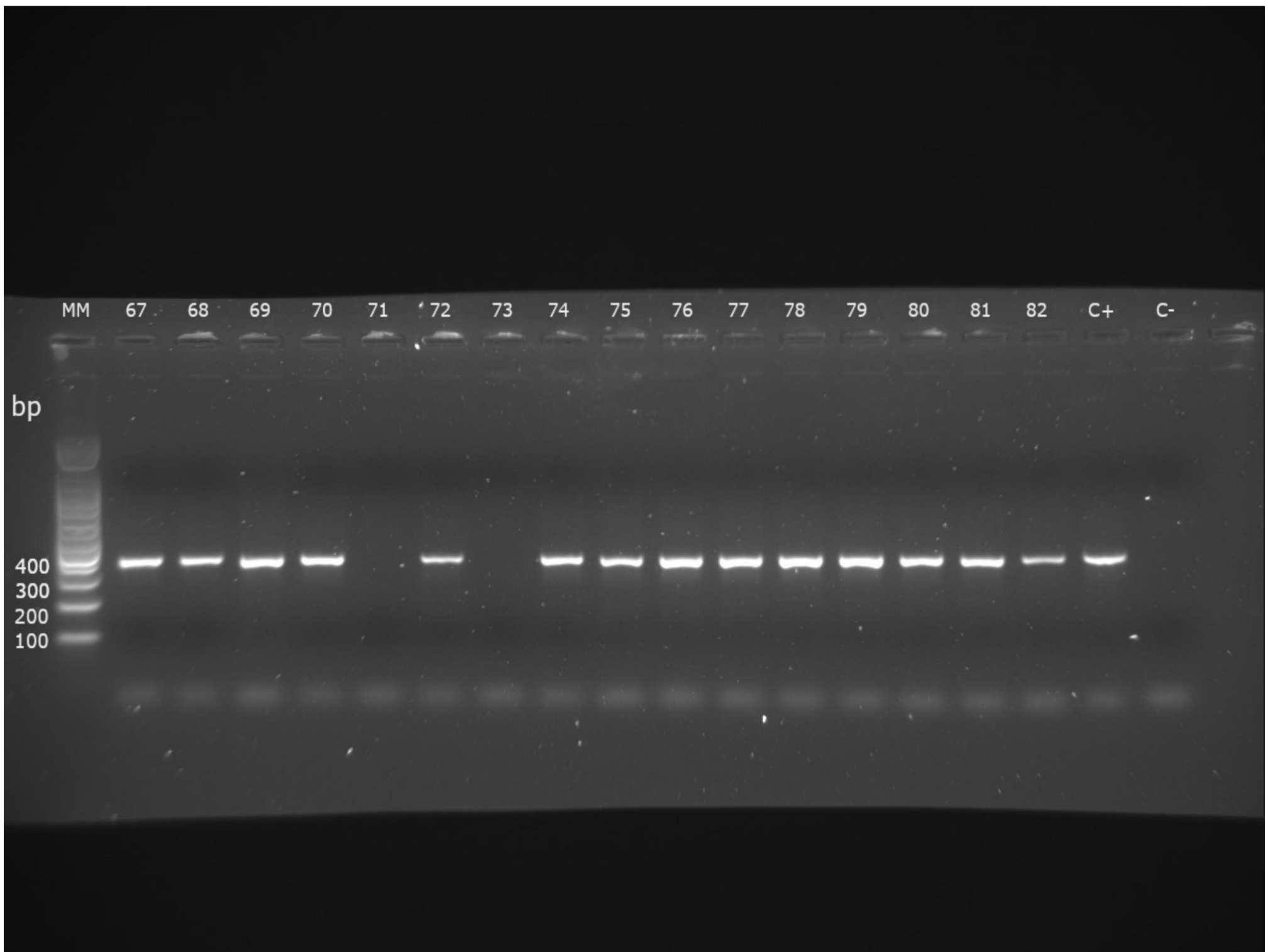

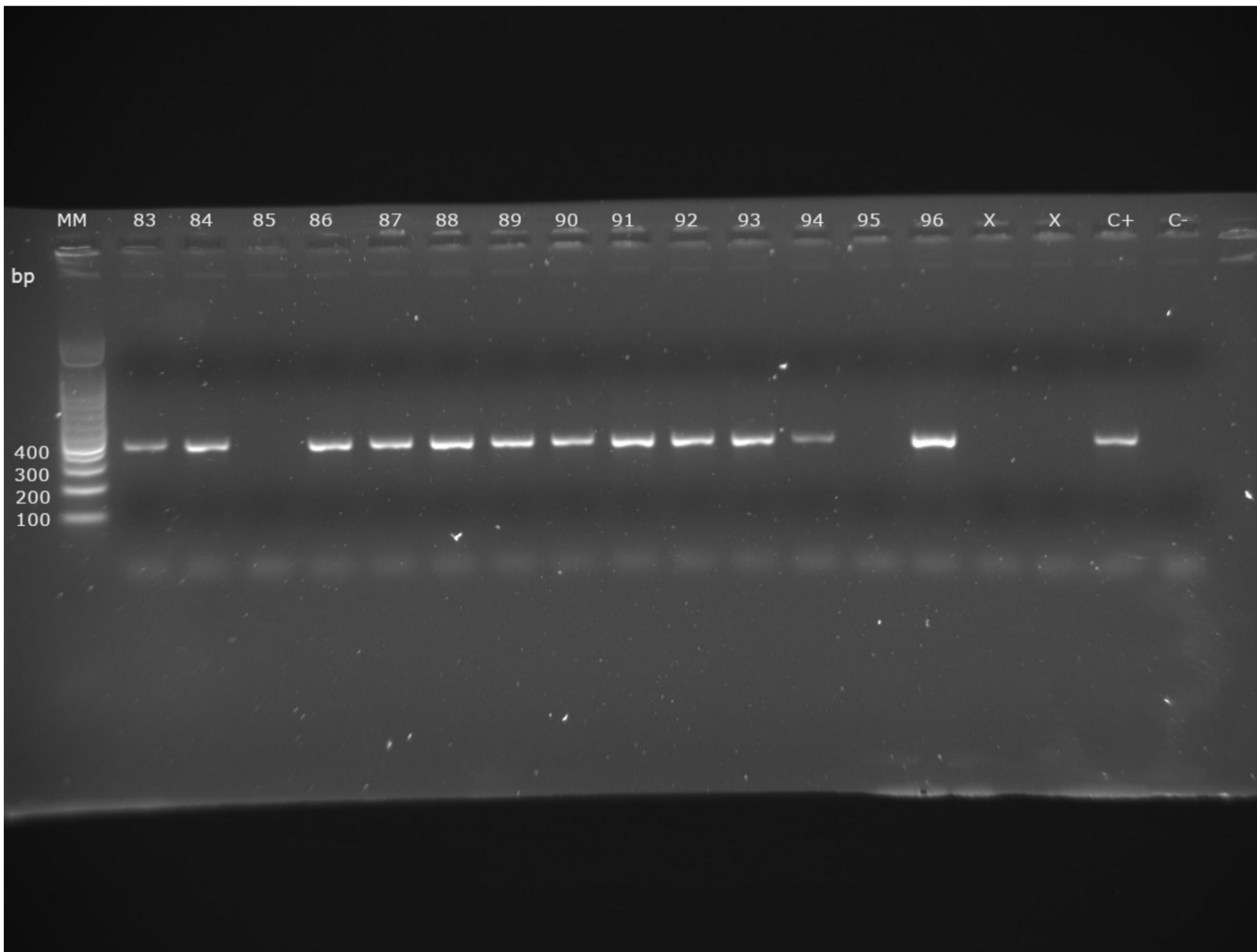

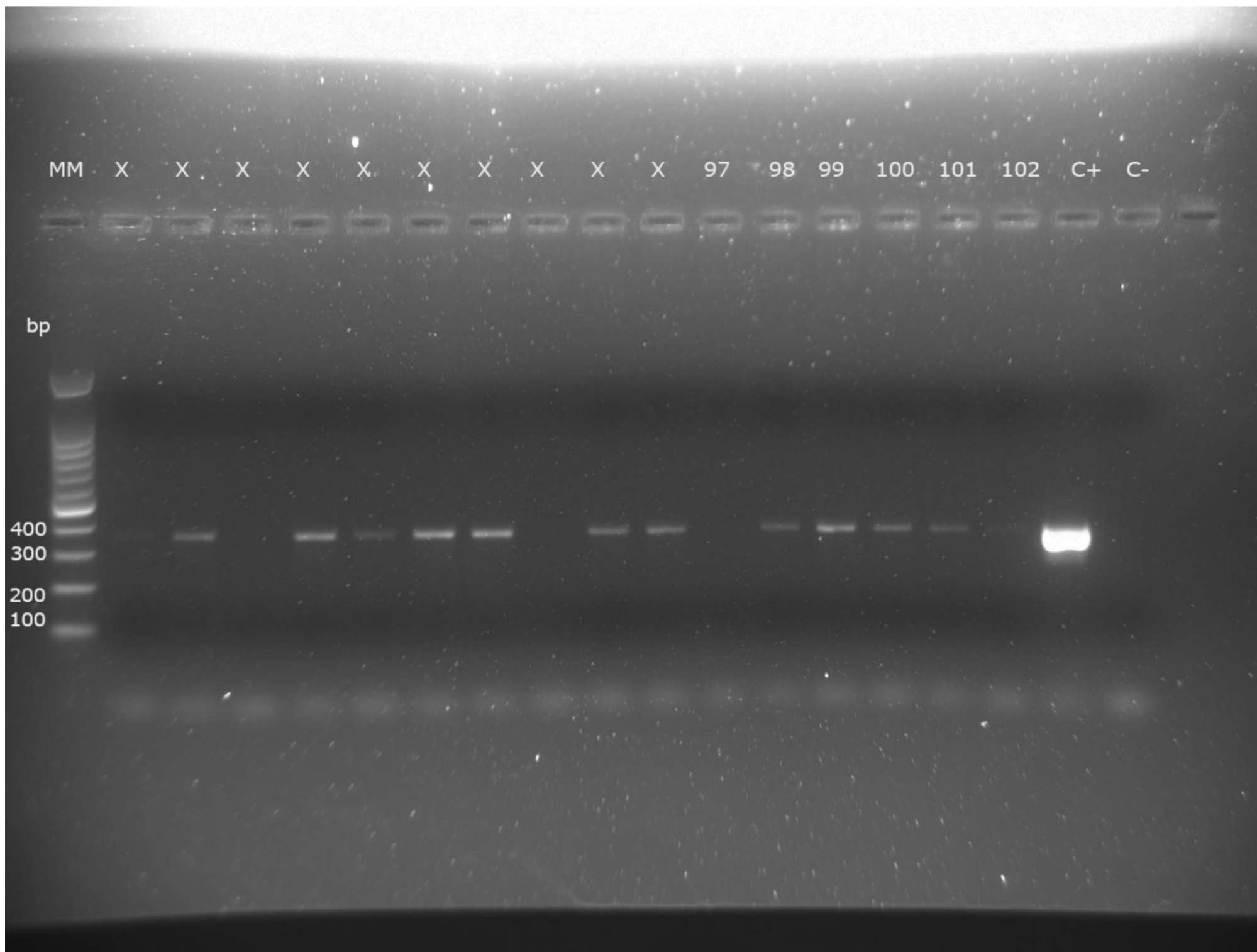

MM 103 104 105 106 107 108 109 110 C+ C-

bp

400  
300  
200  
100

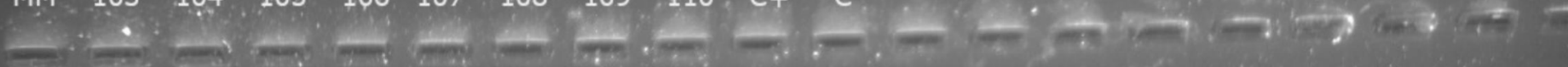

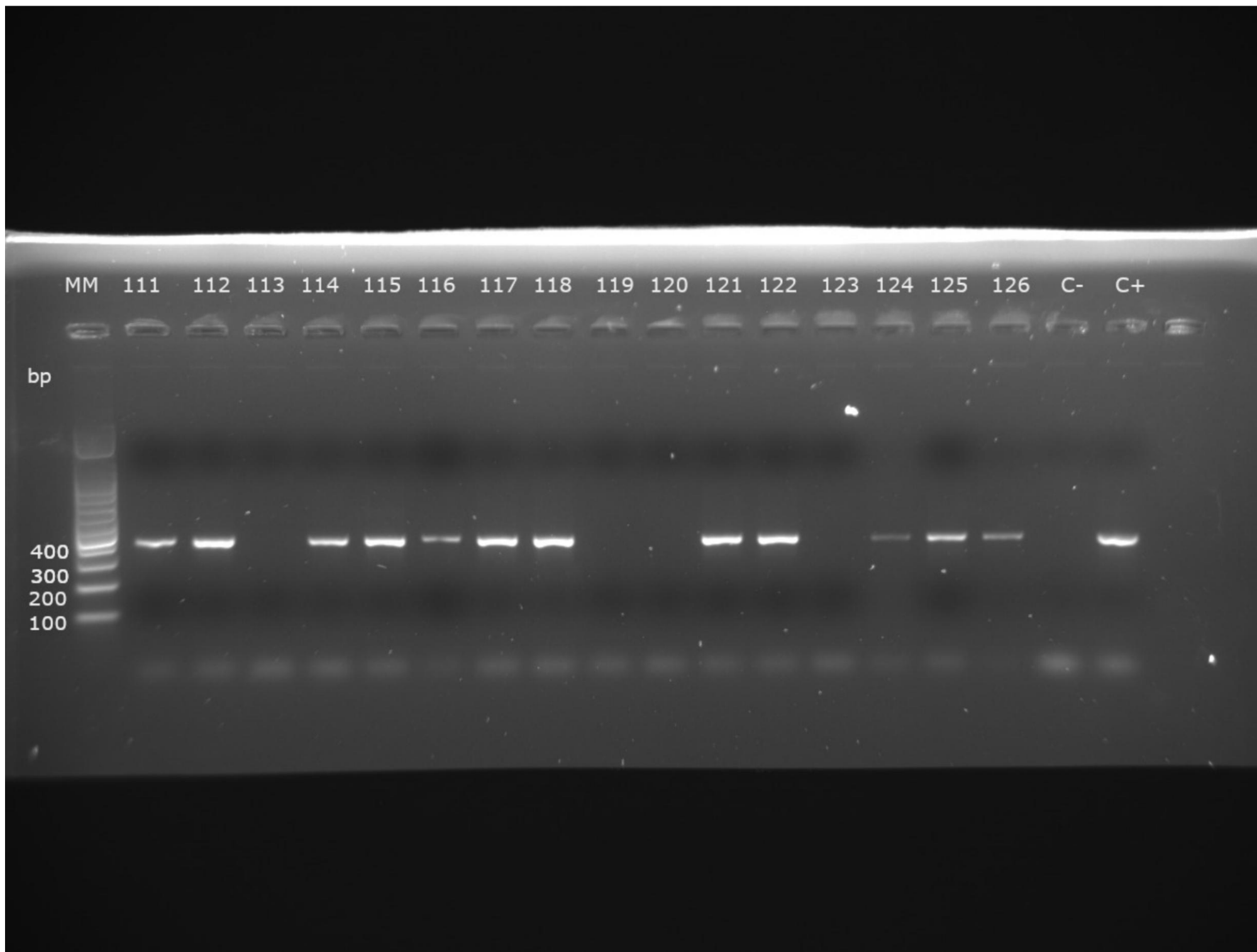

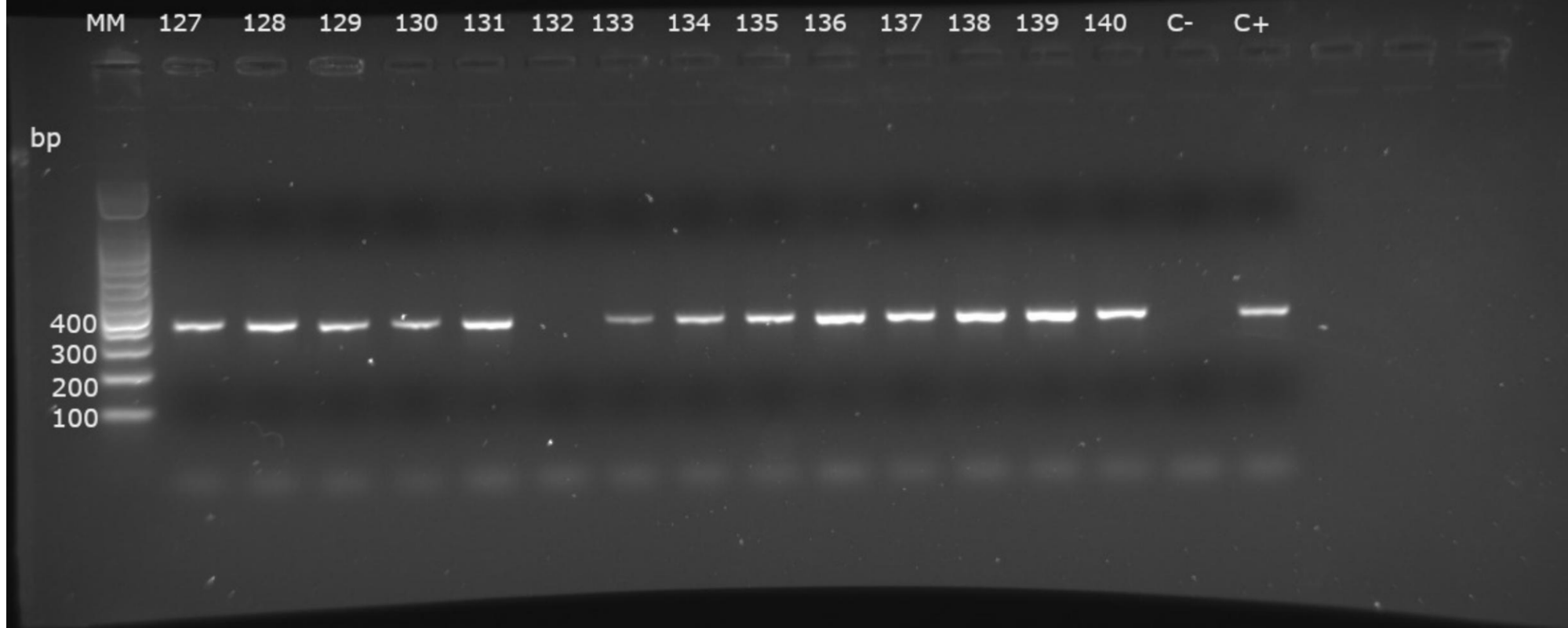

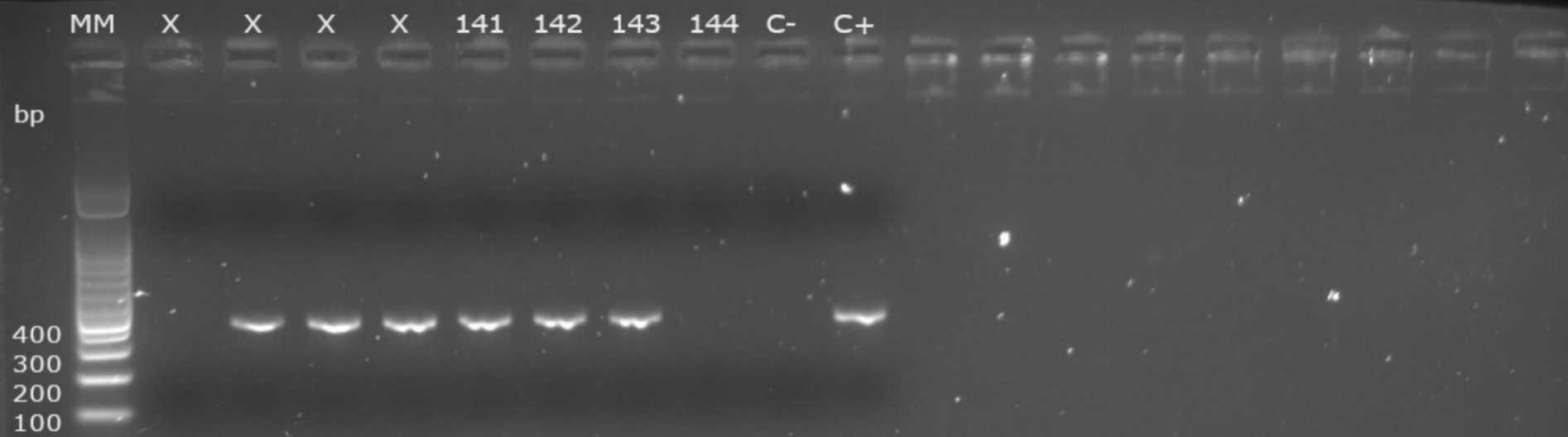

Supplement: S1 Raw Images — Pictures were taken with Gel Imager bio, Vilber Lourmat. Samples were loaded from the left side to the right side. Molecular weight marker was denoted “MM”, positive controls “C+”, negative controls “C-”, and all samples were annotated from 1 to 144 according to their numbering in the database S3 Table. (PDF) [file pone.0332229.s006.pdf]
